# Multiwall and bamboo-like carbon nanotubes from the Allende chondrite: A probable source of asymmetry

**DOI:** 10.1371/journal.pone.0218750

**Published:** 2019-07-01

**Authors:** Hugo I. Cruz-Rosas, Francisco Riquelme, Patricia Santiago, Luis Rendón, Thomas Buhse, Fernando Ortega-Gutiérrez, Raúl Borja-Urby, Doroteo Mendoza, Carlos Gaona, Pedro Miramontes, Germinal Cocho

**Affiliations:** 1 Facultad de Ciencias, Universidad Nacional Autónoma de México, Ciudad Universitaria, Cd. Mx., Mexico; 2 Laboratorio de Sistemática Molecular, Escuela de Estudios Superiores del Jicarero, Universidad Autónoma del Estado de Morelos, Jicarero, Morelos, Mexico; 3 Instituto de Física, Universidad Nacional Autónoma de México, Ciudad Universitaria, Cd. Mx., Mexico; 4 Centro de Investigaciones Químicas, Universidad Autónoma del Estado de Morelos, Cuernavaca, Morelos, Mexico; 5 Instituto de Geología, Universidad Nacional Autónoma de México, Ciudad Universitaria, Cd. Mx., Mexico; 6 Centro de Nanociencias y Micro y Nanotecnologías, Instituto Politécnico Nacional, Zacatenco, Cd. Mx., Mexico; 7 Instituto de Investigaciones en Materiales, Universidad Nacional Autónoma de México, Ciudad Universitaria, Cd. Mx., Mexico; Institute of Materials Science, GERMANY

## Abstract

This study presents multiwall and bamboo-like carbon nanotubes found in samples from the Allende carbonaceous chondrite using high-resolution transmission electron microscopy (HRTEM). A highly disordered lattice observed in this material suggests the presence of chiral domains in it. Our results also show amorphous and poorly-graphitized carbon, nanodiamonds, and onion-like fullerenes. The presence of multiwall and bamboo-like carbon nanotubes have important implications for hypotheses that explain how a probable source of asymmetry in carbonaceous chondrites might have contributed to the enantiomeric excess in soluble organics under extraterrestrial scenarios. This is the first study proving the existence of carbon nanotubes in carbonaceous chondrites.

## Introduction

Carbonaceous chondrites (CCs) show a variety of organic compounds formed in the early solar system [1]. Insoluble organic matter is found as a heterogeneous kerogenic mixture [2,3]. Soluble organic matter shows a diversity of molecules related to terrestrial biological systems [4]. The parent bodies of CCs are thought to have originated when remnant material was accreted to form planetesimals in protoplanetary disks [5]. Furthermore, a variety of post-accretional processes occur on the parent bodies of CCs, including aqueous and thermal alteration [6,7]. The formation of chondrites is a complex and dynamic process with many evolving steps. The Allende meteorite is a Vigarano type chondrite (CV) with a nearly pristine condition (petrologic type 3) [8,9]. It contains macromolecular organic material with high degree of structural organization in its insoluble organic matter, such as polycyclic aromatic hydrocarbons, graphite, nanodiamonds, onion-like structures, and fullerenes [10–13]. To date, no carbon nanotubes (CNTs) have been observed. Nevertheless, evidence such as synthesis of CNTs from opened fullerenes [14] indicates that samples from the Allende chondrite are suitable extraterrestrial material to search for this type of nanostructures.

The composition of organic material in CCs is also a relevant topic in the study of the origin of life. Prebiotic chemistry on Earth has been linked with the chemistry of interplanetary bodies, particularly those processes associated with the origin of biological homochirality, which is a ubiquitous signature for living systems on Earth. Biohomochirality refers to the almost exclusive use of L-amino acids for protein synthesis and the unique use of D-ribose and D-deoxyribose in the structure of RNA and DNA molecules [15]. The homochiral condition is generally described as a fundamental requirement for existence of life [16] and the key process to understand the origin of living systems [17,18]. Thus, the existence of a source of asymmetry operating over the terrestrial chemistry triggering the chiral-imbalance has been hypothesized [19,20]. The molecular asymmetry observed in compounds of CCs has motivated research into the link between extraterrestrial processes and the origin of terrestrial life [21,22]. Such asymmetry has been demonstrated in a kerogen-like component favoring the R-enantiomer in the insoluble organic matter from CCs, including the Allende meteorite [23]. Also, an inorganic matrix was detected in some CCs: measurement by birefringence index shows a bias toward negative values [24]. Furthermore, a suite of sugar derivatives having D-enantiomeric excess (D-EE) ranging from 33% to 82%, have been measured [25]. Methylated α-amino acids has also been found in the soluble organic matter from several CCs with L-enantiomeric excess (L-EE) ranging from 1.2% to 15% [26,27]. This provides evidence that chiral-asymmetric systems are also present outside the Earth. Interestingly, those systems show the same handedness as the corresponding terrestrial compounds: D-EE in sugars and the L-EE in amino acids (AAs).

The chiral asymmetry of L-amino acids and D-sugar derivatives in carbonaceous meteorites is relevant to understanding the physicochemical processes related to the origin of life and its homochirality on Earth. A plausible explanation for the symmetry breaking at interstellar scenarios is the irradiation of circularly polarized light (CPL), which induces asymmetrical photosensitive chemical synthesis or destruction of enantiomers, favoring one handedness [28–30]. This hypothesis is supported by experiments using analogues of interstellar ices, producing L-EE up to 1.34% for amino acids [31]. However, enantiomeric excess measured in an Antarctic chondrite has shown maximum values (up to 60%) [32]. Mechanisms of amplification can be incorporated to attain these high values of chiral-imbalance [27,33,34]. An alternative hypothesis suggests a universal mechanism [27,35] where chiral asymmetry is caused by the energy difference between the enantiomeric couples due to the parity-violation in electroweak interactions [36,37]. It is known that pseudochiral sources can provoke true chirality and enantioselectivity in molecular assemblages [38], such as unidirectional vortices [39] or magnetic fields [40]. The magnetochiral effect (magnetic fields in combination with arbitrarily polarized light) can cause enantioselectivity in chiral macromolecules in interstellar scenarios [41–45]. Additionally, the catalytic properties of mineral and organic surfaces of meteorites have been recognized as a component to induce asymmetric synthesis of soluble organics such as sugars derivatives and AAs [46–48]. In this context, when aggregation processes form surfaces, monomeric constituents can be aligned under influence of magnetochiral asymmetry [44] in a context of emergence of (axial) chirality [49].

Accordingly, CNTs are candidates to influence the syntheses of organics, as demonstrated in the *in silico* studies [50]. These experiments describe a molecular dynamic by which CNTs can absorb the 20 standard AAs. Also, asymmetric influences on the autocatalytic reaction of Soai have been observed by means of CNTs [51,52]. Consequently, CNTs represent a plausible meteoritic-surface capable of driving the physicochemical dynamics in favor of a particular handedness.

In this context, we examine samples of the Allende chondrite using high-resolution transmission electron microscopy (HRTEM) in order to identified CNTs. Our results show that the observed carbonaceous material matches multiwall and bamboo-like CNTs, which show a highly disordered lattice. This indicates that CNTs are candidates to compose a native chiral surface, with implications for the enantiomeric excess in amino acids and sugar derivatives from CCs.

## Materials and methods

### Samples

**Fig 1:**
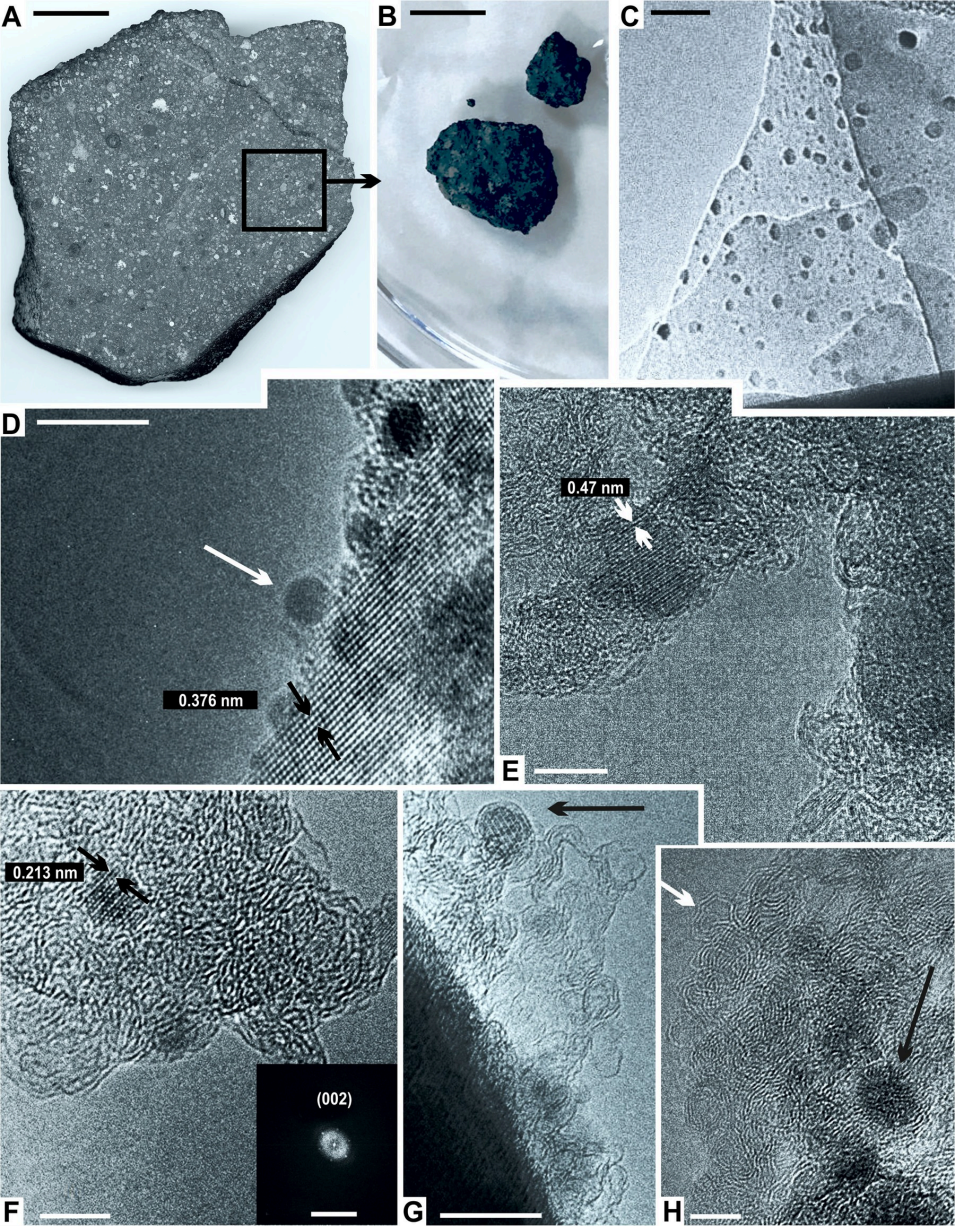
HRTEM analysis of the Allende chondrite. **(A)** Cross-section large CC specimen. Notice the typical black to dark gray color, with abundant rounded chondrules and amorphous calcium-aluminum inclusions. Scale = 4 cm. The black frame indicates the sample extraction area for the HRTEM analysis. **(B)** Small clean samples extracted from the internal structure of the specimen in (A). Scale = 1 cm. **(C)** HRTEM micrograph showing the observed field of the sample. Scale = 20 nm. **(D)** HRTEM micrograph showing a field of carbon with abundant nanoparticles (white arrow), the average fringe distances is 0.376 nm (black arrows). Scale = 5 nm. **(E)** A closer view showing polyhedral graphite zones, constant fringe distances of 0.47 nm (white arrows), and a lattice reflection (101) resembling the tetragonal structure of FeS. Scale = 10 nm. **(F)** Another view of polyhedral graphite. Fringe distances are constant at 0.213 nm (black arrows). Scale = 5 nm. The lattice reflection (002) corresponds to nanodiamonds (bottom right box). Scale = 10 ^1^/nm. **(G)** Another view with onion-like fullerene particles (black arrow). Scale = 10 nm. **(H)** Other onion-like fullerenes (black arrow) embedded in graphite layers (white arrow). Scale = 5 nm.

We used a meteorite specimen of 297.8 g in weight, register number IG-A7, from the Colección Nacional de Meteoritas of the Instituto de Geología, Universidad Nacional Autónoma de México (UNAM), Mexico City (Fig 1A). For transmission electron microscopy (TEM), a ~ 4 cm long fragment of the large specimen were freshly fractured and cut by using a diamond knife in a clean room (Fig 1B). Later, smaller clean fragments (~ 50 mg in total) were extracted from the interior, avoiding contamination from the fusion crust or surface areas (Fig 1B). The resulting crude sample were powdered in an agate mortar and suspended in high-purity ethanol to achieve homogeneous dispersion without agglomerates. For HRTEM analysis, a 5μl pipette was add a portion of that suspension to a lacey carbon grid coated with formvar. Before being used, the TEM grid was carefully inspected in order to rule out any possible contamination.

### HRTEM

High Resolution TEM imaging of chondrite samples was conducted using an Aberration Corrected Cold Field Emission Scanning Transmission Electron Microscope Jeol JEM-ARM200CF at the Centro de Nanociencias y Micro y Nanotecnologías, Instituto Politécnico Nacional, Mexico City. The TEM microscope is equipped with cold field emission gun, Cs-corrector, and high angle annular dark field detector and has ultra-high resolution of 0.72 Å. We utilized an electron beam spot with a condenser aperture of 60 nm at 200 kV for less than 30 seconds. Several locations on individual samples were analyzed. Fast Fourier Transform (FFT) analysis and image processing were applied using the freely available Digital Micrograph (GATAN) software attached at the microscope.

http://dx.doi.org/10.17504/protocols.io.3f4gjqw

## Results

**Fig 2:**
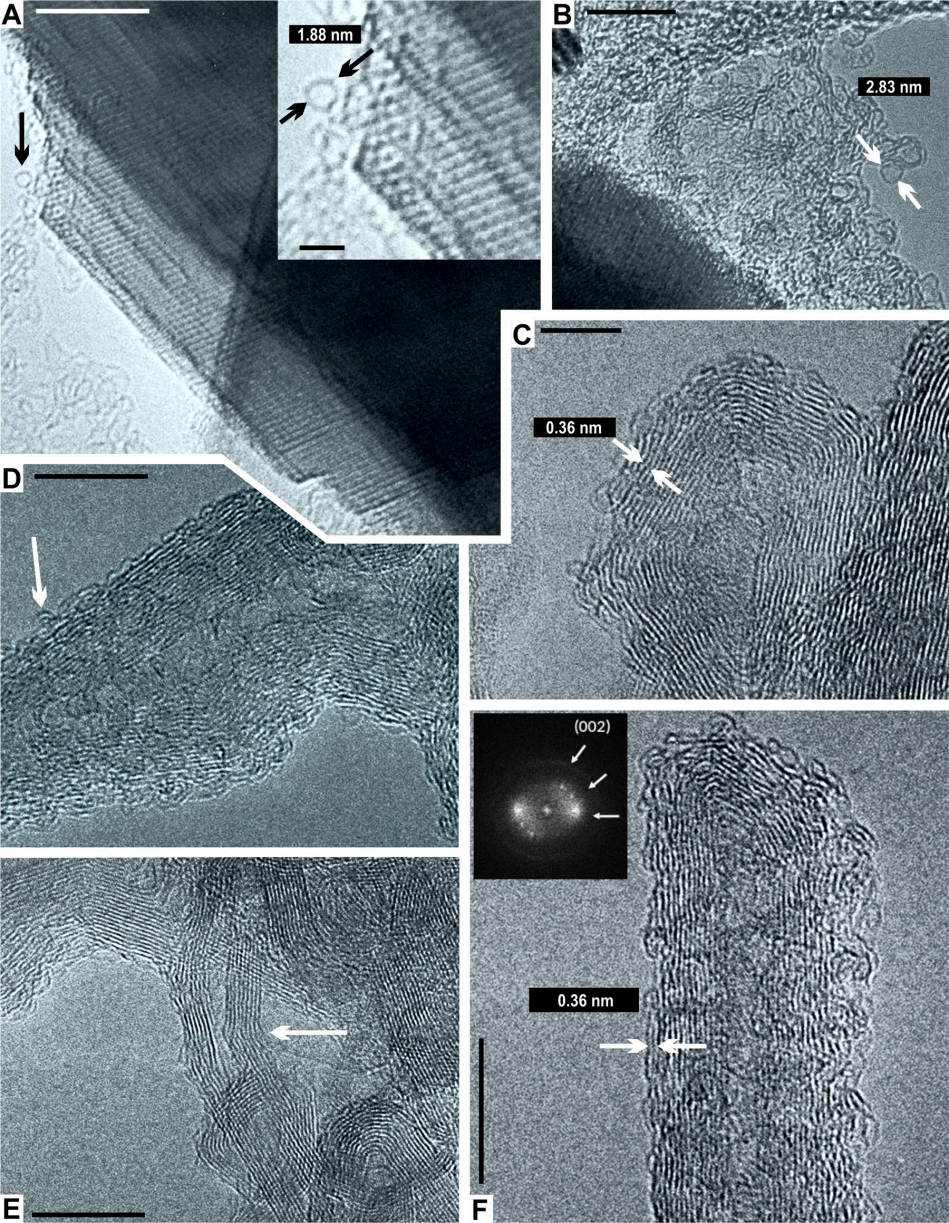
HRTEM analysis of the Allende chondrite. **(A)** A fullerene-like nanosphere (black arrow). Scale = 10 nm. A closer view (top right) shows an approximate size of 1.88 nm (black arrows) that resembles a C60 cage. Scale = 2 nm. **(B)** Another larger fullerene, ~2.83 nm (white arrows). Scale = 10 nm. **(C)** An onion-like fullerene, with constant lattice fringe distances (0.36 nm; white arrows). Scale = 5 nm. **(D)** A field of graphite layers with CNTs (white arrow). Scale = 10 nm. **(E)** Another field of amorphous and polyhedral carbon with several multiwall CNTs. Notice the bending angle close to 36° (white arrow). Scale = 10 nm. **(F)** Closer view showing other multiwall CNTs with highly defective graphene walls. The fringe distances are a constant ~0.36 nm (white arrows), matching the value of turbostratic-stacked graphite. Fast Fourier transform (top left box) shows two split semi-arcs, indicating that the structure is a tip-closed hollow nanotube. Note the dangling carbon bonds on the surface which can make it more chemically reactive. Scale = 10 nm.

The results of HRTEM analysis show a suite of macromolecular components such as poorly-graphitized carbon, amorphous carbon, nanodiamonds, fullerenes, and core-shell structures formed by monocrystalline olivine or pentlandite grains capped by polyhedral graphite layers (Fig 1D–1H). Zones with abundant crystalline nanoparticles no greater than 5 nm in diameter were observed (Fig 1D). These nanoparticles are consistent with a 3C cubic polytype of carbon [53–55]. At higher magnification, the lattice fringes of these nanoparticles were measured. The largest structures show a consistent fringe distance of 0.47 nm and its lattice reflection (101) resembles the tetragonal structure of FeS (Fig 1E). However, the shorter structures show a constant fringe distance at 0.213 nm and their lattice reflection (002) matches nanodiamonds (Fig 1F–1H). In addition, fullerenes and onion-like fullerenes, covered by no more than 10 graphite walls, were observed (Fig 2A–2C). These observations are consistent with previous-published reports [10,12,56,57]. Similar fullerenes and glassy carbon has been observed in the Tagish Lake chondrite [58].

**Fig 3:**
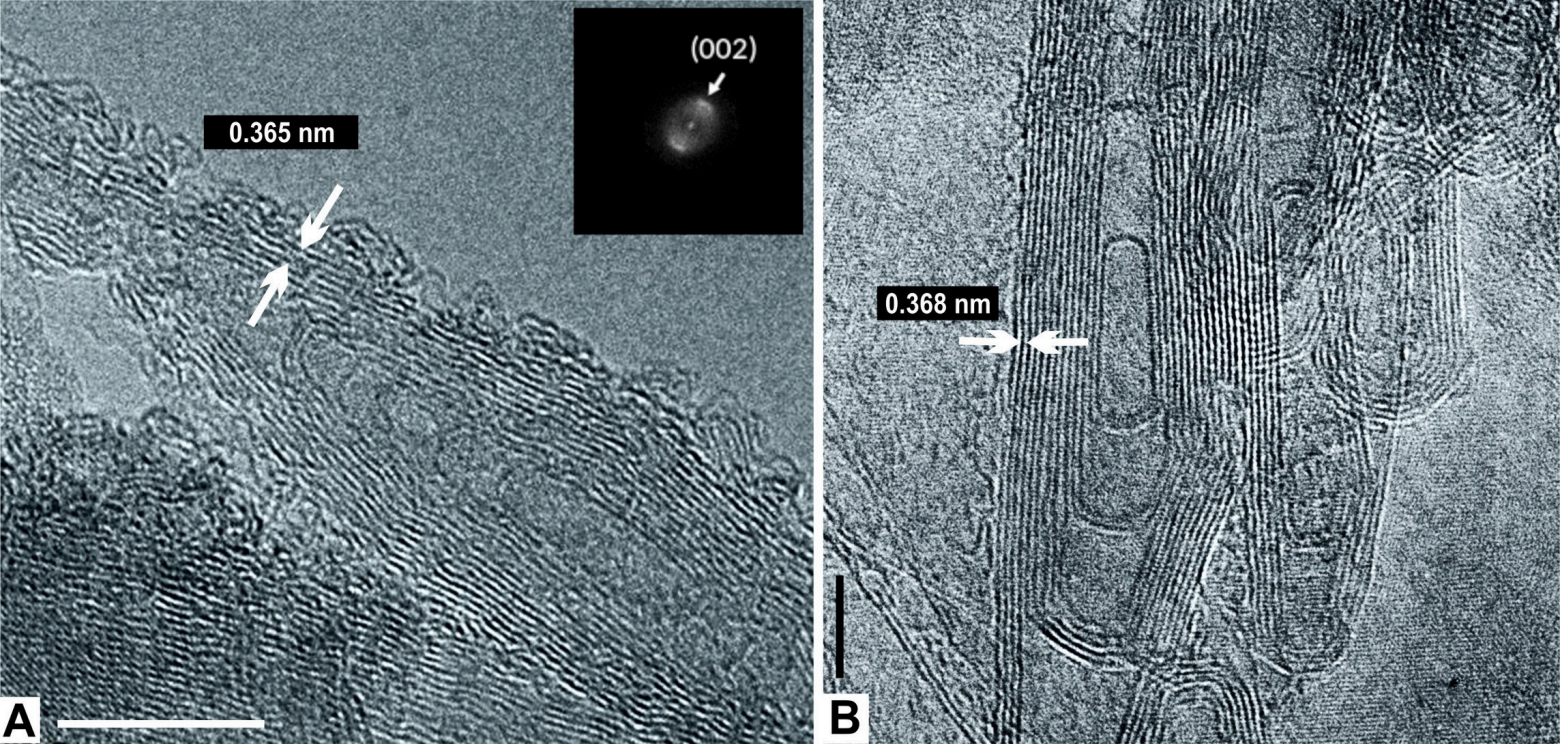
HRTEM analysis of the Allende chondrite. **(A)** View of MWNTs of 10–15 nm in length, with a bamboo-like structure observed. Fringe distances are a constant 0.365 nm (white arrows). Fast Fourier transform (top right box) shows two semi-arcs, indicating that the structure is a hollow nanotube. Scale = 10 nm. **(B)** Another view of bamboo-like MWNTs. Fringe distances are a constant 0.368 nm (white arrows). Scale = 5 nm. For both, note the internal concentric organization in which no single-particle was trapped.

A field of amorphous and polyhedral carbon with multiwall nanotubes (MWNTs) can be seen in Fig 2D–2F. Those nanotubes show a constant fringe distance at 0.36 nm, which is consistent with the turbostratic-stacked graphite [59,60]. It is important to note that interlayer distance is used to characterize allotropes of carbon such as graphite and MWNTs, because the Van der Waals interactions make a strong restriction on the stacked graphene, due to its sp^2^-bonded carbon atoms. The fringe distance for graphite is ~0.34 nm [61,62]. According to this structural analysis, it is possible to demonstrate that our tubular structures are allotropes of carbon. At high magnification, the MWNTs show 14 to 16 highly defective graphene walls. Dangling bonds at the nanotubes surface are also observed (Fig 2F). The roots of the MWNTs are embedded in polyhedral carbon (Fig 2D and 2E). The highly defective graphene walls seen in the nanotubes, suggest that the surface is modified for attachment other molecules or, possibly, that a small indistinct molecule (organic or inorganic) is already attached [63,64]. Fig 2E shows a MWNT with an evident knee and bending angle close to 36°. The knee is produced by pentagon-heptagon defects in the hexagonal graphene lattice [65,66]. The CNTs observed here are consistent with those from the Tagish Lake meteorite [67]. Similar to this study, no particles have been observed inside the CNT of the Tagish Lake meteorite. Also, the root of the nanotube is embedded in the polyhedral carbon [67]. Another field of MWNTs, 10–15 nm in length, with Bamboo-like structure and 6 to 8 concentric graphene-walls, is seen in Fig 3. The Bamboo-like CNTs are composed by hollow compartments with closed tips (Fig 3A) and are embedded in a field of polyhedral graphite (Fig 3A and 3B). No particles can be observed inside the CNTs. This strongly suggests that these large and thick nanotubes grew from open fullerenes under high and fluctuating temperatures [68,69].

The MWNTs observed in this study are not the result of laboratory artifacts caused by inadequate handling of the analysis parameters. Several groups have studied the behavior of MWNTs and other carbon-allotropic structures under *in situ* electron irradiation by HRTEM to experimentally induce the formation of CNTs [70–72]. In contrast, we use an electron beam with a condenser aperture of 60 nm at 200 KeV and an estimated current density of 500 pA/ cm^2^ for less than 30 seconds in a conventional screen area of 270 cm^2^, which is less energetic than the exposition parameters used to experimentally induce the formation of CNTs or other allotropic carbon structures reported in other experiments.

## Discussion

Our results show MWNTs with a highly disordered surface. This allows us to reasonably propose that chiral-domains are present in the lattice. The roll-up vector **Ch** = *n***a**_**1**_ + *m***a**_**2**_ describes the structural properties in a graphene sheet, where *n* and *m* are integers and **a**_**1**_ and **a**_**2**_ are the basal vectors of the graphene lattice [73,74]: when *n* = *m*, the achiral armchair CNTs arise; when *m* = 0, achiral zig-zag CNTs are formed. However, when *n* ≠ *m* and both terms are different from zero, the result is the formation of chiral CNTs [75]. That sort of disordered surface is the basis of the expectation that the roll-up vector **Ch** can describe chiral arrangements at the local level in the MWNTs. It is very likely that the chiral surface of MWNTs can form by aggregation processes from carbon-vapor under asymmetric magnetochiral influence in the parent body in CCs, as seen in the processes of fullerene formation by deposition of carbon atoms [12]. Thus, open fullerenes act as nucleation point for vapor-gas carbon atoms like the model of Rümmeli and coworkers [14]. Under these conditions, the CNTs might have grown without catalytic particle. Thereby, we can expect that MWNT formation could have been biased due to magnetochiral influence on the parent body of CCs, bearing in mind that the associations between light and magnetic fields are common in the cosmos [76]. To emphasize this, we take into account that the Allende chondrite, among other CCs, has a unidirectional magnetization record that is explained by the existence of a core dynamo in the parent body [77]. Although the CCs have experienced thermal and aqueous alteration afterwards, however, allotropes of carbon are prevalent, with minor changes since protoplanetary nebula times [78].

The multiwall structure observed in CNTs and fullerenes makes reference to a common formation process, where the three-dimensional surface of graphite is covered by the consecutive layers. It has been suggested that the graphitization of nanodiamonds during thermal events gives rise to onion-like fullerenes over periods of millions of years at temperatures below 300°C [10]. Another scenario suggests that the fullerenes are formed from the humps of the graphite layers, and through thermal processes (i.e. pyrolysis), they are opened [14]. Therefore, those thermally opened fullerenes may serve as nucleation points for growth of CNTs. In this study, nanodiamonds and fullerenes are consistently found in the Allende chondrite [56,57]. The Allende parent body has experienced fluctuating thermal metamorphism in the protoplanetary nebula over long time scales [10,79,80]. However, the low level of graphitization, characteristic of petrologic type 3 chondrites, allowed the formation of graphene, nanodiamond, fullerenes, carbon onions [10,56–58] and CNTs, consistent with the temperatures of the inner region of the protosolar nebula [59,79,80]. Macromolecular organic compounds from the Allende chondrite have experienced temperatures between 2000 to 2200°C [12]. Consequently, both multiwall and bamboo-like CNTs might have formed from open-fullerenes during thermal processes with high fluctuating temperatures in carbon-rich environment. Accordingly, the MWNTs could be formed during the accretion of the meteorite parent body in a protosolar scenario. The septum in the bamboo-like CNTs might have formed by deformation of the lattice, either by means of thermal fluctuations [81] or by incorporating other atoms to the carbon sheet, such as N, O, or H [82], as shown by its highly disordered surfaces [63].

The previous scenario is only about the formation of a meteoritic organic surface composed by CNTs, which could be formed under asymmetric magnetochiral influence. The subsequent events of thermal and aqueous alterations on soluble organics could have taken place in presence of the MWNTs. This meteoritic carbon allotropes can be related with the enantiomeric excess in sugar-derivatives and amino acids of CCs. The proposed mechanism to this is that MWNTs, as a native chiral-surface, can work to bias the synthesis of soluble organics towards one enantiomer, or, alternatively, maintaining its enantioselective absorption. These scenario could be the source for constant chiral asymmetry on AAs and sugar-derivatives, that can be amplified by a subsequent mechanism. A difficulty for this theoretical approach is the absence, until now, of observations of chiral asymmetry in soluble organics in Allende meteorite. Nonetheless, our confirmation of the existence of MWNTs in Allende chondrite, makes very possible its presence in another carbonaceous chondrites with confirmed enantiomeric excess in soluble organics.

## Conclusions

We present the first record of multiwall and bamboo-like CNTs in samples from the Allende chondrite. Bamboo-like CNTs are the first observation for extraterrestrial material. Our results has theoretical implications with the enantiomeric excess of amino acids and sugar derivatives that can be found in carbonaceous chondrites.

## Supporting information

S1 Fig Specimen of the Allende chondrite. Cross-section of the used large specimen.
(TIF)

S2 Fig Small pieces from the Allende chondrite. Extracted pieces from the specimen.
(TIF)

S3 Fig HRTEM image of the Allende chondrite. Micrograph of the observed field.
(TIF)

S4 Fig HRTEM image of the Allende chondrite. Field with abundant nanoparticles.
(TIF)

S5 Fig HRTEM image of the Allende chondrite. Field with polyhedral graphite.
(TIF)

S6 Fig HRTEM image of the Allende chondrite. Field with polyhedral graphite.
(TIF)

S7 Fig HRTEM image of the Allende chondrite. Field with onion-like fullerene.
(TIF)

S8 Fig HRTEM image of the Allende chondrite. Field with onion-like fullerene.
(TIF)

S9 Fig HRTEM image of the Allende chondrite. Field with fullerene-like nanosphere.
(TIF)

S10 Fig HRTEM image of the Allende chondrite. Closer view of fullerene-like nanosphere.
(TIF)

S11 Fig HRTEM image of the Allende chondrite. Large fullerenes.
(TIF)

S12 Fig HRTEM image of the Allende chondrite. Onion-like fullerene.
(TIF)

S13 Fig HRTEM image of the Allende chondrite. Multiwall carbon nanotube.
(TIF)

S14 Fig HRTEM image of the Allende chondrite. Bent multiwall carbon nanotube.
(TIF)

S15 Fig HRTEM image of the Allende chondrite. Large multiwall carbon nanotube.
(TIF)

S16 Fig HRTEM image of the Allende chondrite. Fast Fourier transform from carbon nanotube in S15 Fig.
(TIF)

S17 Fig HRTEM image of the Allende chondrite. Bamboo-like multiwall carbon nanotube.
(TIF)

S18 Fig HRTEM image of the Allende chondrite. Fast Fourier transform from bamboo-like carbon nanotube in S17 Fig.
(TIF)

S19 Fig HRTEM image of the Allende chondrite. Bamboo-like multiwall carbon nanotube.
(TIF)

## References

[1] Ehrenfreund P, Cami J. Cosmic carbon chemistry: from the interstellar medium to the early Earth. Cold Spring Harbor perspectives in biology. 2010 10.1101/cshperspect.a002097 20554702 PMC2982172

[2] Hayes JM. Organic constituents of meteorites-a review. Geochim Cosmochim Acta. 1967; 10.1016/0016-7037(67)90019-1

[3] Aoki T, Akai J. Carbon materials in Antarctic and nonAntarctic carbonaceous chondrites: high-resolution transmission electron microscopy. J Mineral Petrol Sci. 2008; 10.2465/jmps.070301

[4] Pizzarello S. The chemistry of life’s origin: A carbonaceous meteorite perspective. Accounts of Chemical Research. 2006 10.1021/ar050049f 16618090

[5] Maurette M. Classification of meteorites and micrometeorites Micrometeorites and the Mysteries of Our Origins. Springer; 2006 pp. 54–71.

[6] Krot AN, Petaev MI, Scott ERD, Choi BG, Zolensky ME, Keil K. Progressive alteration in CV3 chondrites: More evidence for asteroidal alteration. Meteorit Planet Sci. 1998; 10.1111/j.1945-5100.1998.tb01713.x

[7] Glavin DP, Callahan MP, Dworkin JP, Elsila JE. The effects of parent body processes on amino acids in carbonaceous chondrites. Meteorit Planet Sci. 2010; 10.1111/j.1945-5100.2010.01132.x

[8] Sánchez-Rubio G. Allende, una piedra extraordinaria. Boletín Mineral. 1992;5: 38–45.

[9] Lozano-Santa Cruz R. La clasificación de los meteoritos. Boletín Mineral. 1992;5: 56–64. Available: https://biblat.unam.mx/es/revista/boletin-de-mineralogia

[10] Le Guillou C, Rouzaud JN, Bonal L, Quirico E, Derenne S, Remusat L. High resolution TEM of chondritic carbonaceous matter: Metamorphic evolution and heterogeneity. Meteorit Planet Sci. 2012; 10.1111/j.1945-5100.2012.01336.x

[11] Huss GR. Meteoritic Nanodiamonds: Messengers from the Stars. Elements. 2005; 10.2113/gselements.1.2.97

[12] Harris PJF, Vis RD. High-resolution transmission electron microscopy of carbon and nanocrystals in the Allende meteorite. Proc R Soc A Math Phys Eng Sci. 2003; 10.1098/rspa.2003.1125

[13] Ehrenfreund P, Foing BH. Fullerenes and cosmic carbon. Science. 2010 10.1126/science.1194855 20813945

[14] Rümmeli MH, Bachmatiuk A, Börrnert F, Schäffel F, Ibrahim I, Cendrowski K, et al Synthesis of carbon nanotubes with and without catalyst particles. Nanoscale Research Letters. 2011 10.1186/1556-276X-6-303 21711812 PMC3211370

[15] Kauffman SA. Approaches to the Origin of Life on Earth. Life. 2011; 10.3390/life1010034 25382055 PMC4187126

[16] Carroll JD. A new definition of life. Chirality. 2009; 10.1002/chir.20590 18571800

[17] Wu M, Walker SI, Higgs PG. Autocatalytic Replication and Homochirality in Biopolymers: Is Homochirality a Requirement of Life or a Result of It? Astrobiology. 2012; 10.1089/ast.2012.0819 22931294

[18] Cruz-Rosas HI, Riquelme F, Maldonado M, Cocho G. Critical role of spatial information from chiral-asymmetric peptides in the earliest occurrence of life. Int J Astrobiol. Cambridge University Press; 2017;16: 28–39.

[19] Bonner WA. The origin and amplification of biomolecular chirality. Orig Life Evol Biosph. 1991; 10.1007/BF01809580 1758688

[20] Breslow R, Cheng Z-L. On the origin of terrestrial homochirality for nucleosides and amino acids. Proc Natl Acad Sci. 2009; 10.1073/pnas.0904350106 19478058 PMC2695116

[21] Bonner WA. Chirality and life. Orig Life Evol Biosph. Springer; 1995;25: 175–190. 11536669 10.1007/BF01581581

[22] Pizzarello S, Shock E, Ferris J. Carbonaceous Chondrite Meteorites: the Chronicle of a Potential Evolutionary Path between Stars and Life. Orig Life Evol Biosph. 2017; 10.1007/s11084-016-9530-1 28078499

[23] Kawasaki T, Hatase K, Fujii Y, Jo K, Soai K, Pizzarello S. The distribution of chiral asymmetry in meteorites: An investigation using asymmetric autocatalytic chiral sensors. Geochim Cosmochim Acta. 2006; 10.1016/j.gca.2006.09.019

[24] Arteaga O, Canillas A, Crusats J, El-Hachemi Z, Jellison GE, Llorca J, et al Chiral biases in solids by effect of shear gradients: A speculation on the deterministic origin of biological homochirality. Orig Life Evol Biosph. 2010; 10.1007/s11084-009-9184-3 19924561

[25] Cooper G, Rios AC. Enantiomer excesses of rare and common sugar derivatives in carbonaceous meteorites. Proc Natl Acad Sci. 2016; 10.1073/pnas.1603030113 27247410 PMC4914185

[26] Pizzarello S. Molecular Asymmetry in Prebiotic Chemistry: An Account from Meteorites. Life. 2016; 10.3390/life6020018 27089368 PMC4931455

[27] Burton A, Berger E. Insights into abiotically-generated amino acid enantiomeric excesses found in meteorites. Life. Multidisciplinary Digital Publishing Institute; 2018;8: 14. 10.3390/life8020014 PMC6027462 29757224

[28] Bonner WA, Rubenstein E. Supernovae, neutron stars and biomolecular chirality. BioSystems. 1987; 10.1016/0303-2647(87)90025-6 3580540

[29] Rubenstein E, Bonner WA, Noyes HP, Brown GS. Supernovae and life. Nature. Springer; 1983;306: 118.

[30] Bonner WA. Terrestrial and extraterrestrial sources of molecular homochirality. Orig Life Evol Biosph. 1991; 10.1007/BF01808311

[31] De Marcellus P, Meinert C, Nuevo M, Filippi JJ, Danger G, Deboffle D, et al Non-racemic amino acid production by ultraviolet irradiation of achiral interstellar ice analogs with circularly polarized light. Astrophys J Lett. 2011; 10.1088/2041-8205/727/2/L27 21907648

[32] Pizzarello S, Schrader DL, Monroe AA, Lauretta DS. Large enantiomeric excesses in primitive meteorites and the diverse effects of water in cosmochemical evolution. Proc Natl Acad Sci. 2012; 10.1073/pnas.1204865109 22778439 PMC3409747

[33] Rivera Islas J, Micheau JC, Buhse T. Kinetic analysis of self-replicating peptides: Possibility of chiral amplification in open systems. Orig Life Evol Biosph. 2004; 10.1023/B:ORIG.0000043115.95561.23 15573499

[34] Soai K, Kawasaki T. Asymmetric autocatalysis with amplification of chirality. Top Curr Chem. 2008; 10.1007/128-2007-138 16676331

[35] Pavlov V, Klabunovskii E. Homochirality Origin in Nature: Possible Versions. Curr Org Chem. 2014; 10.2174/13852728113179990033

[36] Laerdahl JK, Schwerdtfeger P, Quiney HM. Theoretical analysis of parity-violating energy differences between the enantiomers of chiral molecules. Phys Rev Lett. 2000; 10.1103/PhysRevLett.84.3811 11019212

[37] Quack M. How important is parity violation for molecular and biomolecular chirality? Angewandte Chemie—International Edition. 2002 10.1002/anie.200290005 12481315

[38] Barron LD. True and False Chirality and Absolute Asymmetric Synthesis. J Am Chem Soc. 1986; 10.1021/ja00278a029

[39] Ribó JM, Crusats J, Sagués F, Claret J, Rubires R. Chiral sign induction by vortices during the formation of mesophases in stirred solutions. Science (80-). 2001; 10.1126/science.1060835 11408653

[40] Micali N, Engelkamp H, Van Rhee PG, Christianen PCM, Scolaro LM, Maan JC. Selection of supramolecular chirality by application of rotational and magnetic forces. Nat Chem. 2012; 10.1038/nchem.1264 22354434

[41] Thiemann W, Jarzak U. A new idea and experiment related to the possible interaction between magnetic field and stereoselectivity. Orig Life. Springer; 1981;11: 85–92. 7231984 10.1007/BF00928000

[42] Wagnière G, Meier A. Difference in the absorption coefficient of enantiomers for arbitrarily polarized light in a magnetic field: A possible source of chirality in molecular evolution. Experientia. 1983; 10.1007/BF01943122

[43] Rikken GLJA, Raupach E. Observation of magneto-chiral dichroism. Nature. 1997; 10.1038/37323

[44] Rikken GLJA, Raupach E. Enantioselective magnetochiral photochemistry. Nature. 2000; 10.1038/35016043 10879530

[45] Kitagawa Y, Segawa H, Ishii K. Magneto-chiral dichroism of organic compounds. Angew Chemie—Int Ed. 2011; 10.1002/anie.201101809 21796747

[46] Saladino R, Botta G, Delfino M, Di Mauro E. Meteorites as catalysts for prebiotic chemistry. Chem—A Eur J. 2013; 10.1002/chem.201303690 24307356

[47] Rotelli L, Trigo-Rodríguez JM, Moyano-Cambero CE, Carota E, Botta L, Di Mauro E, et al The key role of meteorites in the formation of relevant prebiotic molecules in a formamide/water environment. Sci Rep. Nature Publishing Group; 2016;6: 38888 10.1038/srep38888 27958316 PMC5153646

[48] Saladino R, Botta L, Di Mauro E. The prevailing catalytic role of meteorites in formamide prebiotic processes. Life. Multidisciplinary Digital Publishing Institute; 2018;8: 6. 10.3390/life8010006 PMC5871938 29470412

[49] González-Campo A, Amabilino DB. Biomolecules at interfaces: chiral, naturally. Biochirality. Springer; 2013 pp. 109–156. 10.1007/128_2012_405 23460199

[50] Az’Hari S, Ghayeb Y. Effect of chirality, length and diameter of carbon nanotubes on the adsorption of 20 amino acids: A molecular dynamics simulation study. Mol Simul. 2014; 10.1080/08927022.2013.812210

[51] Rance GA, Miners SA, Chamberlain TW, Khlobystov AN. The effect of carbon nanotubes on chiral chemical reactions. Chem Phys Lett. Elsevier; 2013;557: 10–14.

[52] Hitosugi S, Matsumoto A, Kaimori Y, Iizuka R, Soai K, Isobe H. Asymmetric autocatalysis initiated by finite single-wall carbon nanotube molecules with helical chirality. Org Lett. 2014; 10.1021/ol403384q 24417337

[53] Daulton TL, Eisenhour DD, Bernatowicz TJ, Lewis RS, Buseck PR. Genesis of presolar diamonds: Comparative high-resolution transmission electron microscopy study of meteoritic and terrestrial nano-diamonds. Geochim Cosmochim Acta. 1996; 10.1016/S0016-7037(96)00223-2

[54] Santiago P, Camacho-Bragado GA, Marin-Almazo M, Murgich J, José-Yacaman M. Diamond polytypes in mexican crude oil. Energy and Fuels. 2004; 10.1021/ef034049c

[55] Hu M, Tian F, Zhao Z, Huang Q, Xu B, Wang LM, et al Exotic cubic carbon allotropes. J Phys Chem C. 2012; 10.1021/jp3064323

[56] Becker L, Bunch TE. Fullerenes, fulleranes and polycyclic aromatic hydrocarbons in the Allende meteorite. Meteorit Planet Sci. 1997; 10.1111/j.1945-5100.1997.tb01292.x 11540421

[57] Becker L, Bunch TE, Allamandola LJ. Higher fullerenes in the Allende meteorite. Nature. 1999 10.1038/22250 10421363

[58] Becker L, Poreda RJ, Nuth JA, Ferguson FT, Liang F, Billups WE. Fullerenes in meteorites and the nature of planetary atmospheres Natural fullerenes and related structures of elemental carbon. Springer; 2006 pp. 95–121.

[59] Vis RD, Mrowiec A, Kooyman PJ, Matsubara K, Heymann D. Microscopic search for the carrier phase Q of the trapped planetary noble gases in Allende, Leoville and Vigarano. Meteorit Planet Sci. 2002; 10.1111/j.1945-5100.2002.tb01036.x

[60] Hashimoto A, Suenaga K, Urita K, Shimada T, Sugai T, Bandow S, et al Atomic correlation between adjacent graphene layers in double-wall carbon nanotubes. Phys Rev Lett. 2005; 10.1103/PhysRevLett.94.045504 15783570

[61] Dubey P, Sonkar SK, Majumder S, Tripathi KM, Sarkar S. Isolation of water soluble carbon nanotubes with network structure possessing multipodal junctions and its magnetic property. RSC Adv. 2013; 10.1039/c3ra22933e

[62] Sonkar SK, Tripathi KM, Sarkar S. Ferromagnetic Behaviour of Anthropogenic Multi-Walled Carbon Nanotubes Trapped in Spider Web Indoor. J Nanosci Nanotechnol. 2014; 10.1166/jnn.2014.8524 24745259

[63] Wepasnick KA, Smith BA, Bitter JL, Howard Fairbrother D. Chemical and structural characterization of carbon nanotube surfaces. Analytical and Bioanalytical Chemistry. 2010 10.1007/s00216-009-3332-5 20052581

[64] Muhulet A, Miculescu F, Voicu SI, Schütt F, Thakur VK, Mishra YK. Fundamentals and scopes of doped carbon nanotubes towards energy and biosensing applications. Materials Today Energy. 2018 10.1016/j.mtener.2018.08.005

[65] Schaefer H-E. Carbon Nanostructures Carbon nanostructures–Tubes, Graphene graphene, Fullerenes fullerenes, Wave-Particle Duality wave-particle duality. Nanoscience. Springer; 2010 pp. 209–266.

[66] Fejes D, Hernádi K. A review of the properties and CVD synthesis of coiled carbon nanotubes. Materials (Basel). 2010; 10.3390/ma3042618

[67] Garvie LAJ, Buseck PR. Nanosized carbon-rich grains in carbonaceous chondrite meteorites. Earth Planet Sci Lett. 2004; 10.1016/j.epsl.2004.05.024

[68] Andrews R, Jacques D, Qian D, Rantell T. Multiwall carbon nanotubes: Synthesis and application. Acc Chem Res. 2002; 10.1021/ar010151m 12484788

[69] Alekseev NI. On the morphology of carbon nanotubes growing from catalyst particles: Formulation of the model. Phys Solid State. Springer; 2006;48: 1605–1615.

[70] Harris PJF. Carbon nanotubes and other graphitic structures as contaminants on evaporated carbon films. J Microsc. 1997; 10.1046/j.1365-2818.1997.1930754.x

[71] Harris PJF. Carbonaceous contaminants on support films for transmission electron microscopy. Carbon N Y. 2001; 10.1016/S0008-6223(00)00195-0

[72] Banhart F, Li JX, Krasheninnikov A V. Carbon nanotubes under electron irradiation: Stability of the tubes and their action as pipes for atom transport. Phys Rev B—Condens Matter Mater Phys. 2005; 10.1103/PhysRevB.71.241408

[73] Odom TW, Huang JL, Kim P, Ouyang M, Lieber CM. Scanning tunneling microscopy and spectroscopy studies of single wall carbon nanotubes. J Mater Res. 1998; 10.1557/JMR.1998.0331

[74] Meyer RR, Friedrichs S, Kirkland AI, Sloan J, Hutchison JL, Green MLH. A composite method for the determination of the chirality of single walled carbon nanotubes. J Microsc. Wiley Online Library; 2003;212: 152–157. 14629564 10.1046/j.1365-2818.2003.01240.x

[75] Dresselhaus MS, Dresselhaus G, Saito R. Physics of carbon nanotubes. Carbon N Y. 1995; 10.1016/0008-6223(95)00017-8

[76] Barron LD. Chemistry: Chirality, magnetism and light. Nature. Nature Publishing Group; 2000;405: 895 10.1038/35016183 10879516

[77] Elkins-Tanton LT, Weiss BP, Zuber MT. Chondrites as samples of differentiated planetesimals. Earth Planet Sci Lett. 2011; 10.1016/j.epsl.2011.03.010

[78] Sephton MA. Organic compounds in carbonaceous meteorites. Natural Product Reports. 2002 10.1039/b103775g 12137279

[79] Krot AN, Yurimoto H, Hutcheon ID, Libourel G, Chaussidon M, Tissandier L, et al Type C Ca, Al-rich inclusions from Allende: Evidence for multistage formation. Geochim Cosmochim Acta. 2007; 10.1016/j.gca.2006.09.019

[80] Amelin Y, Krot A. Pb isotopic age of the Allende chondrules. Meteorit Planet Sci. 2007; 10.1111/j.1945-5100.2007.tb00577.x

[81] Louchev OA. Formation mechanism of pentagonal defects and bamboo-like structures in carbon nanotube growth mediated by surface diffusion. Physica Status Solidi (A) Applied Research. 2002 10.1002/1521-396X(200210)193:3<585::AID-PSSA585>3.0.CO;2-Y

[82] Sumpter BG, Meunier V, Romo-Herrera JM, Cruz-Silva E, Cullen DA, Terrones H, et al Nitrogen-mediated carbon nanotube growth: Diameter reduction, metallicity, bundle dispersability, and bamboo-like structure formation. ACS Nano. 2007; 10.1021/nn700143q 19206689

