# Supplementary figures and images for: Multiwall and bamboo-like carbon nanotubes from the Allende chondrite: A probable source of asymmetry
Source: PLoS One. 2019 Jul 1;14(7):e0218750. doi: 10.1371/journal.pone.0218750 (PMC6602194; doi:10.1371/journal.pone.0218750)

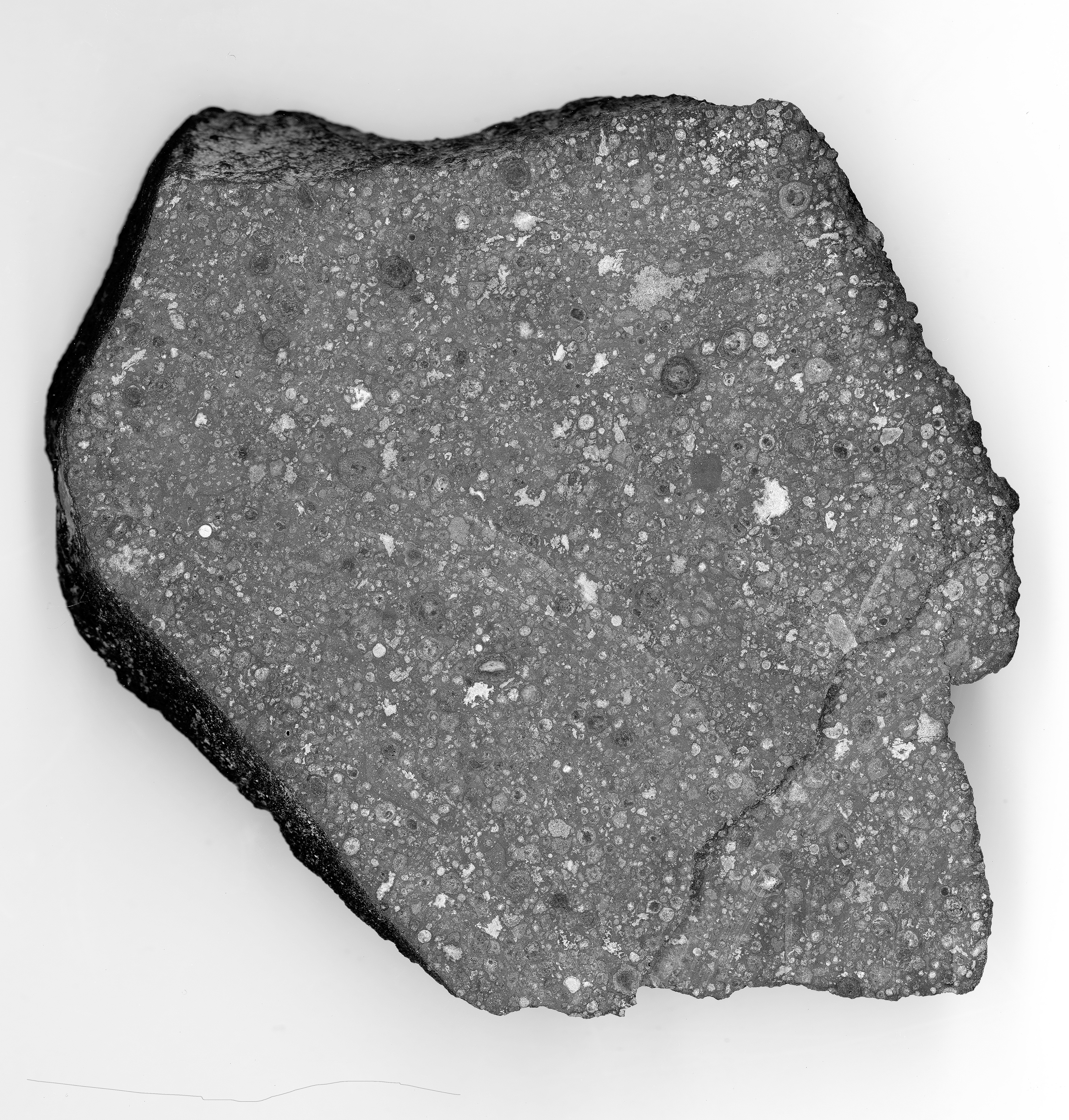

Supplement: S1 Fig — Cross-section of the used large specimen. (TIF) [file pone.0218750.s001.tif]

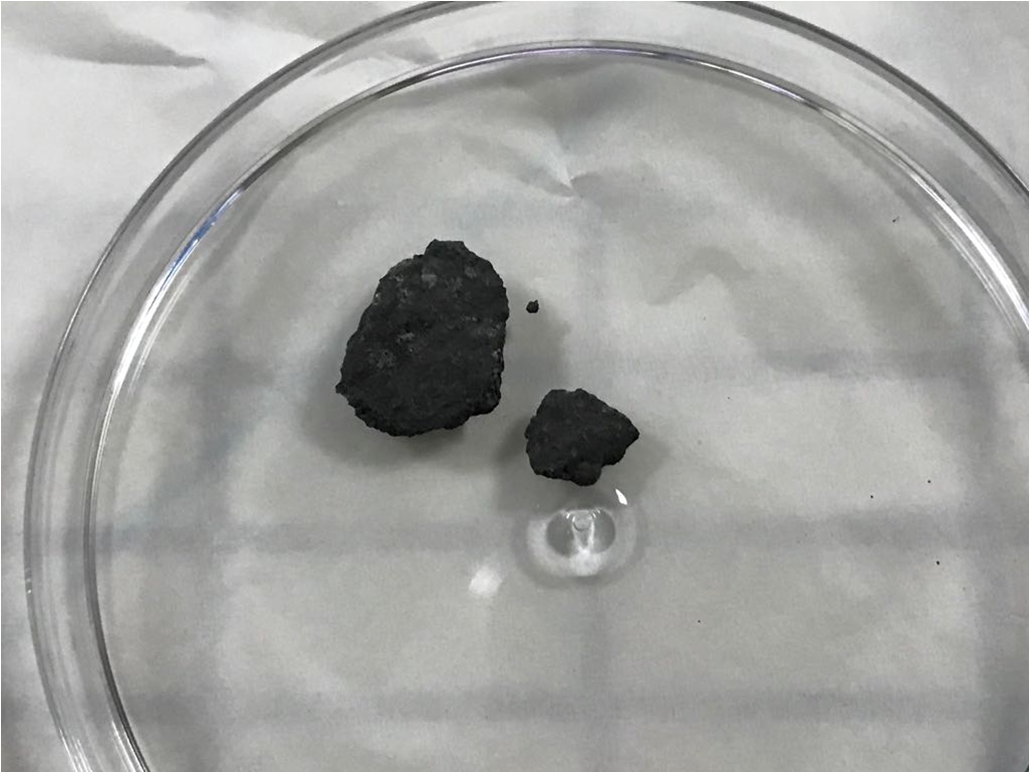

Supplement: S2 Fig — Extracted pieces from the specimen. (TIF) [file pone.0218750.s002.tif]

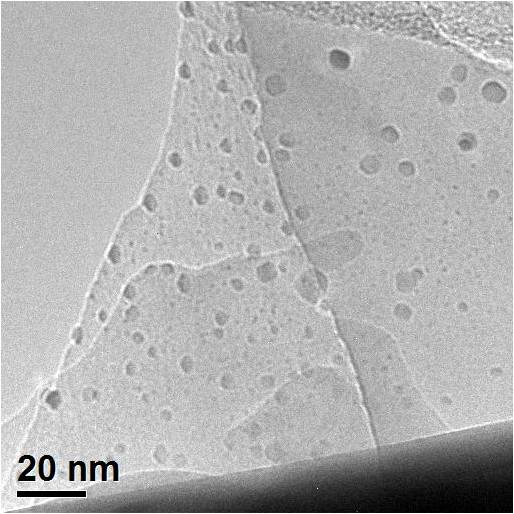

Supplement: S3 Fig — Micrograph of the observed field. (TIF) [file pone.0218750.s003.tif]

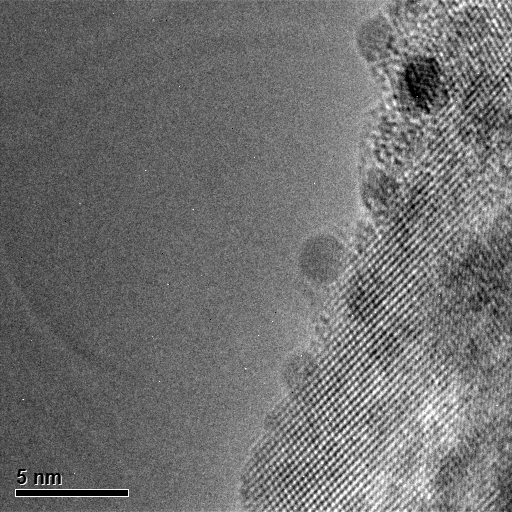

Supplement: S4 Fig — Field with abundant nanoparticles. (TIF) [file pone.0218750.s004.tif]

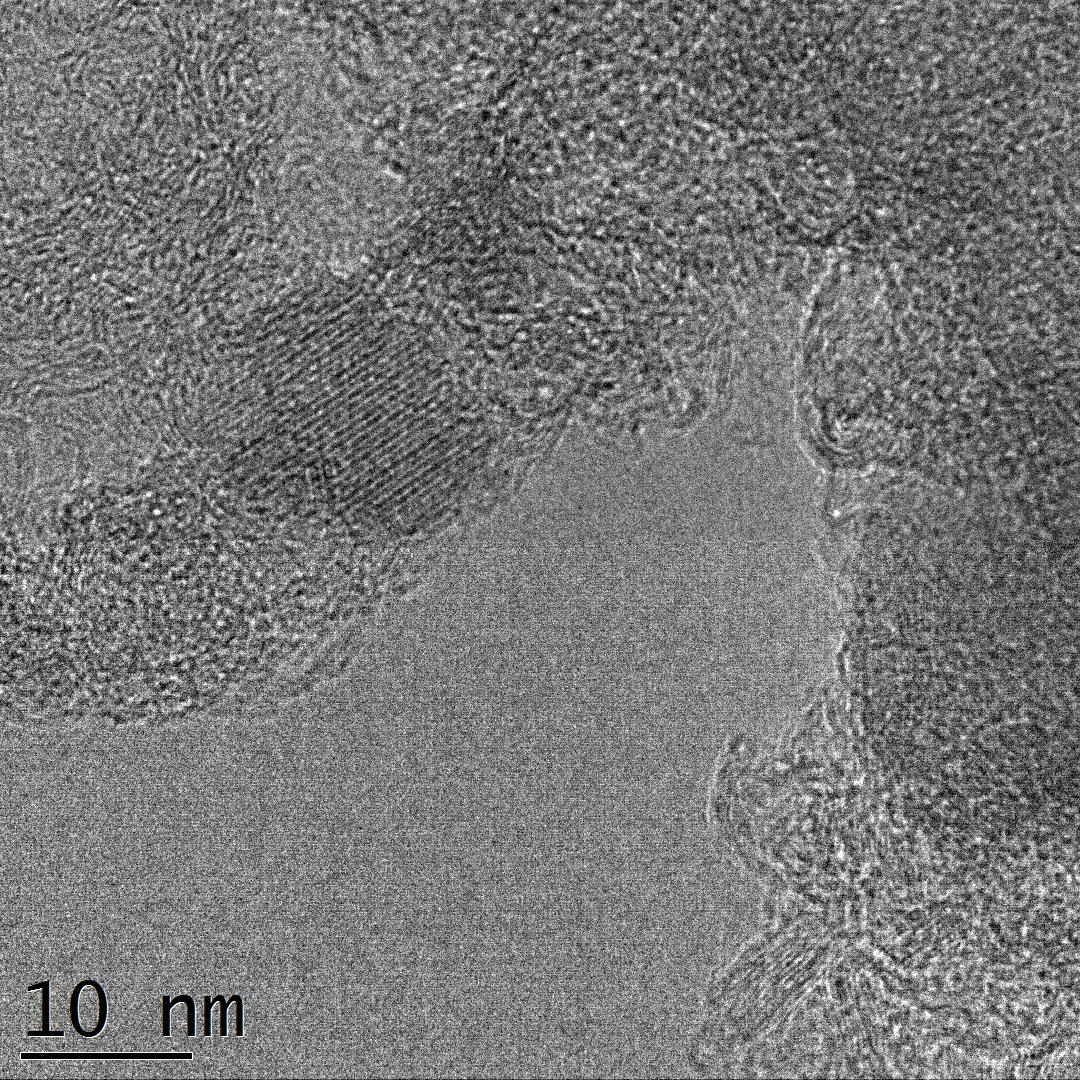

Supplement: S5 Fig — Field with polyhedral graphite. (TIF) [file pone.0218750.s005.tif]

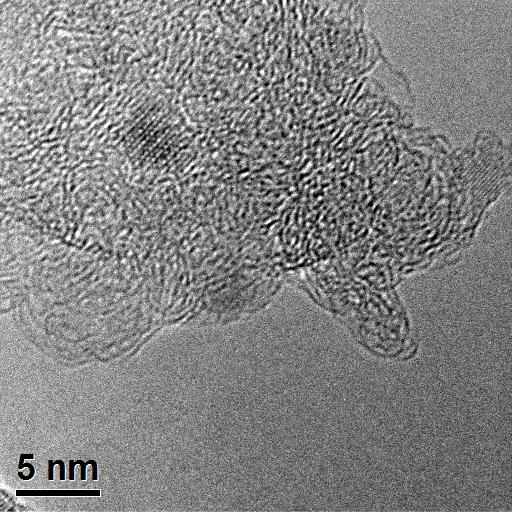

Supplement: S6 Fig — Field with polyhedral graphite. (TIF) [file pone.0218750.s006.tif]

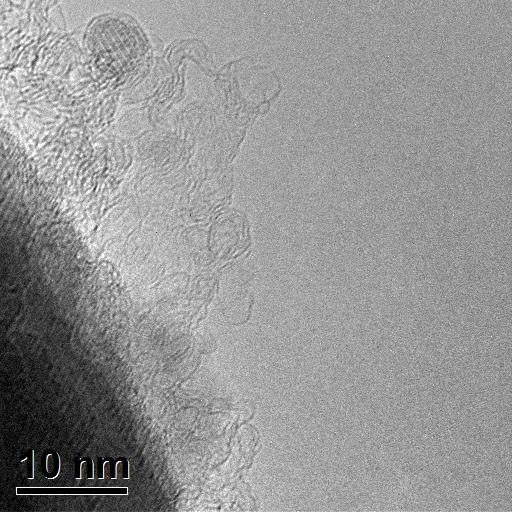

Supplement: S7 Fig — Field with onion-like fullerene. (TIF) [file pone.0218750.s007.tif]

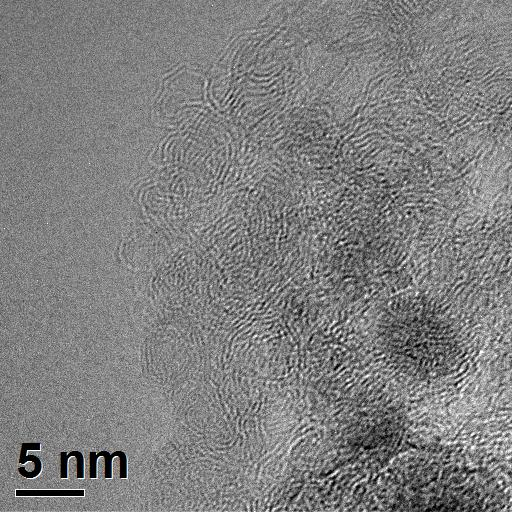

Supplement: S8 Fig — Field with onion-like fullerene. (TIF) [file pone.0218750.s008.tif]

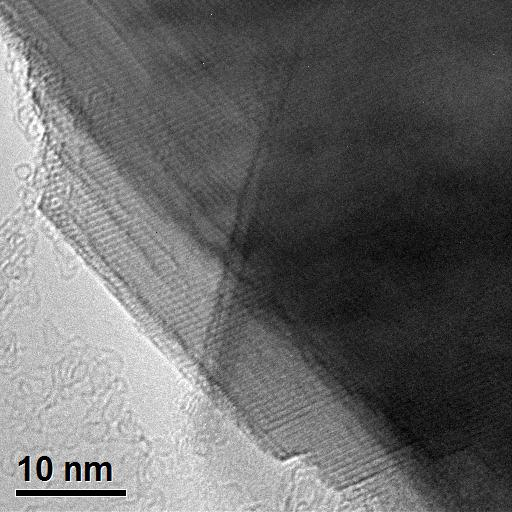

Supplement: S9 Fig — Field with fullerene-like nanosphere. (TIF) [file pone.0218750.s009.tif]

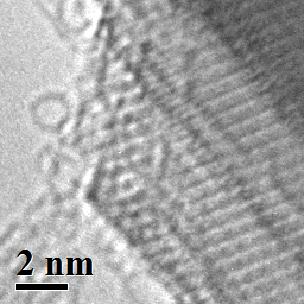

Supplement: S10 Fig — Closer view of fullerene-like nanosphere. (TIF) [file pone.0218750.s010.tif]

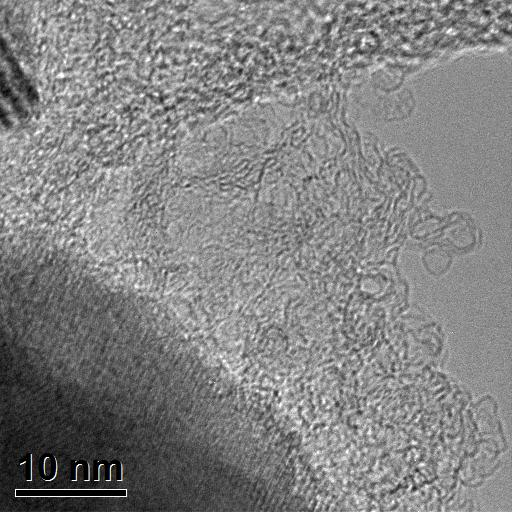

Supplement: S11 Fig — Large fullerenes. (TIF) [file pone.0218750.s011.tif]

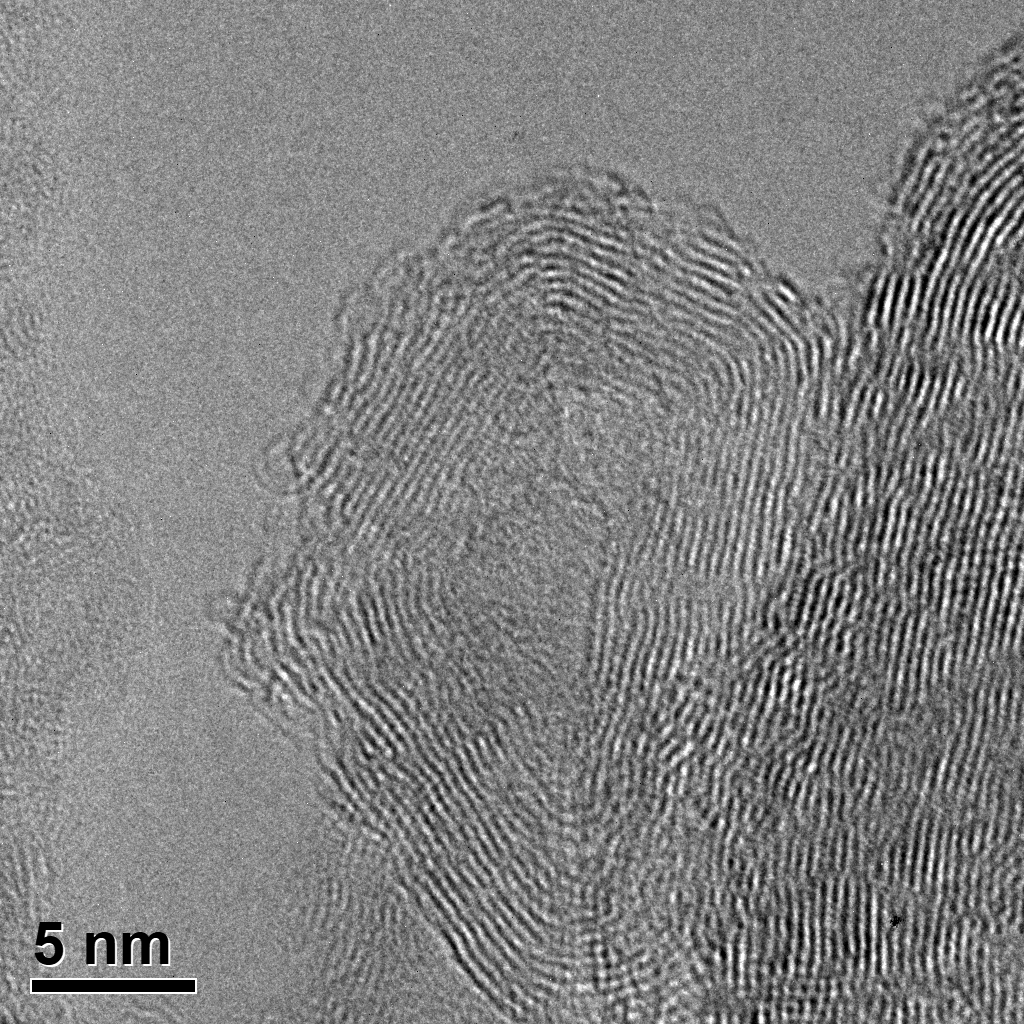

Supplement: S12 Fig — Onion-like fullerene. (TIF) [file pone.0218750.s012.tif]

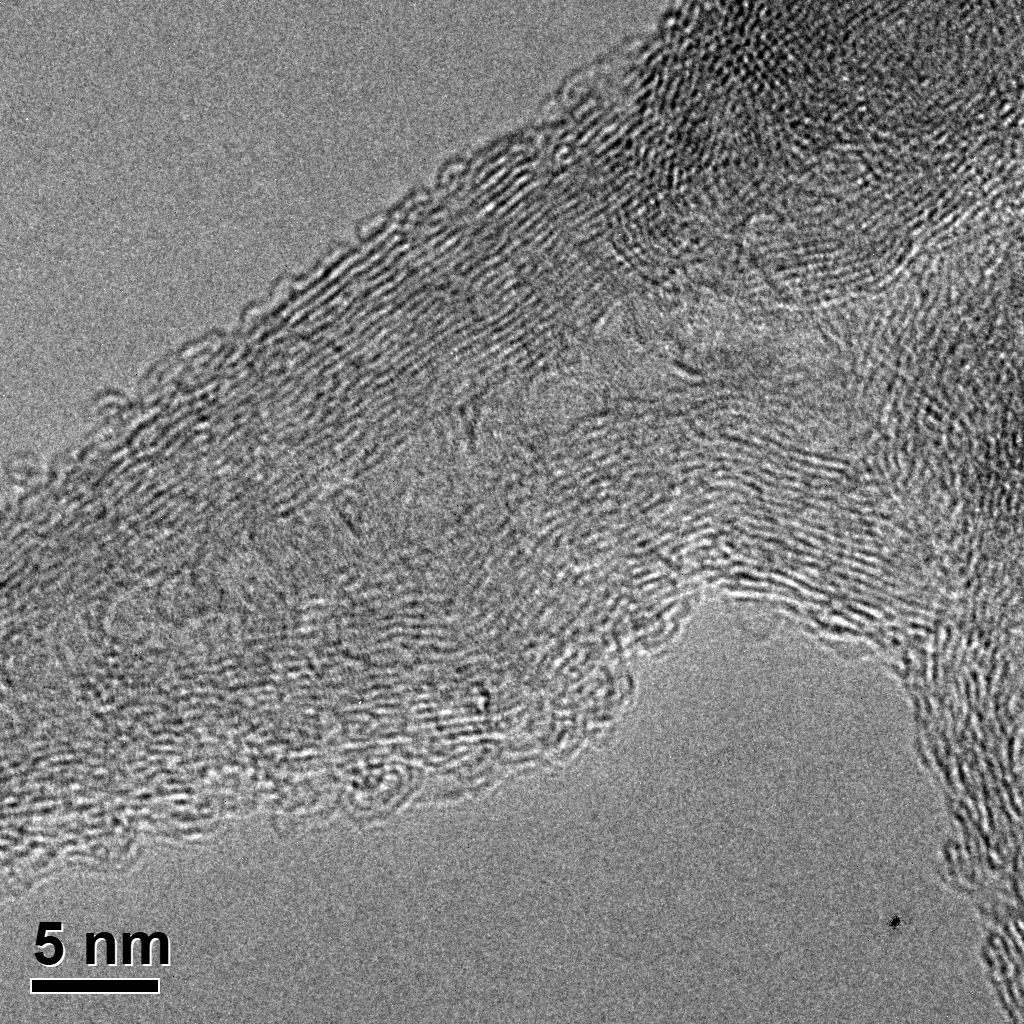

Supplement: S13 Fig — Multiwall carbon nanotube. (TIF) [file pone.0218750.s013.tif]

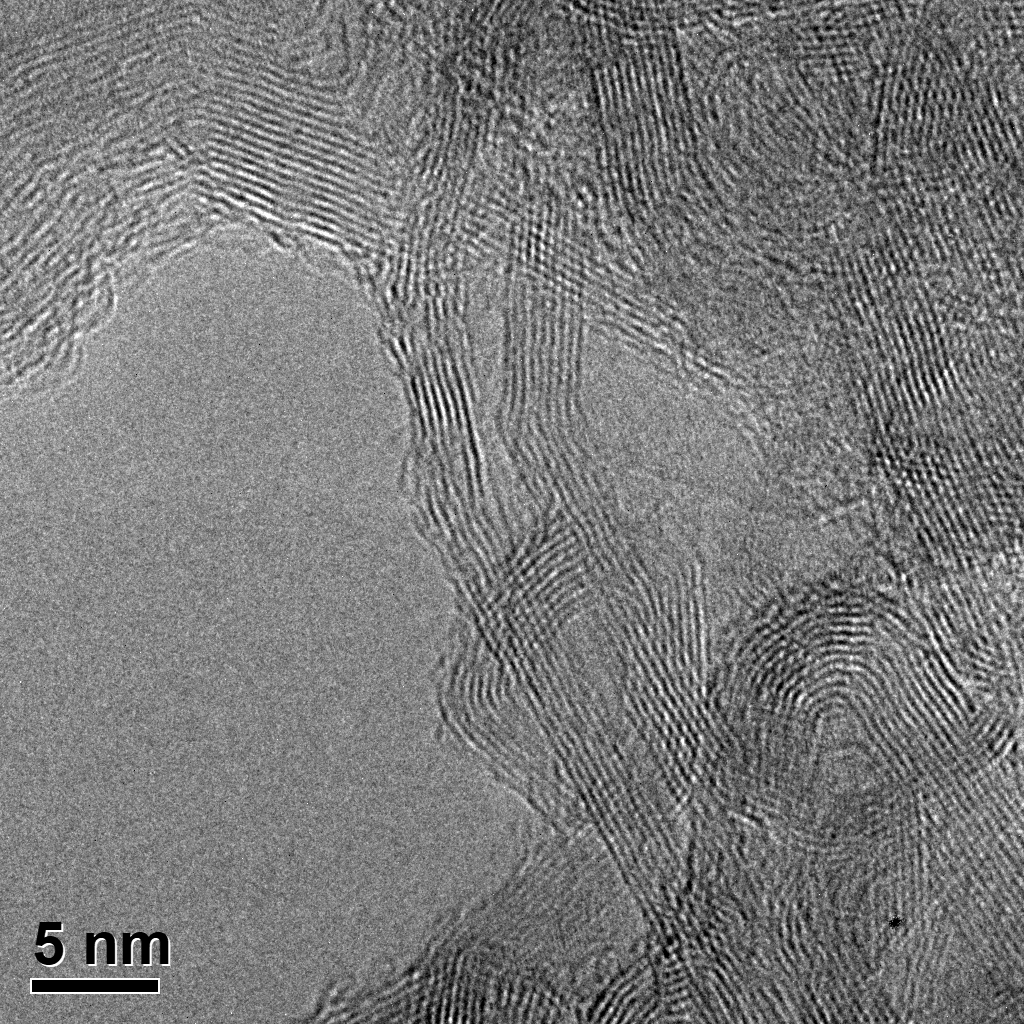

Supplement: S14 Fig — Bent multiwall carbon nanotube. (TIF) [file pone.0218750.s014.tif]

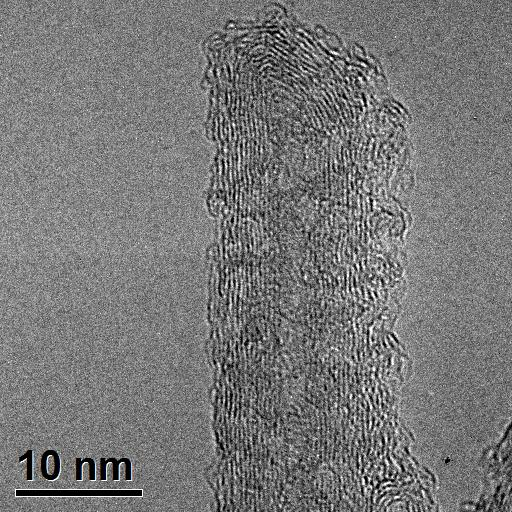

Supplement: S15 Fig — Large multiwall carbon nanotube. (TIF) [file pone.0218750.s015.tif]

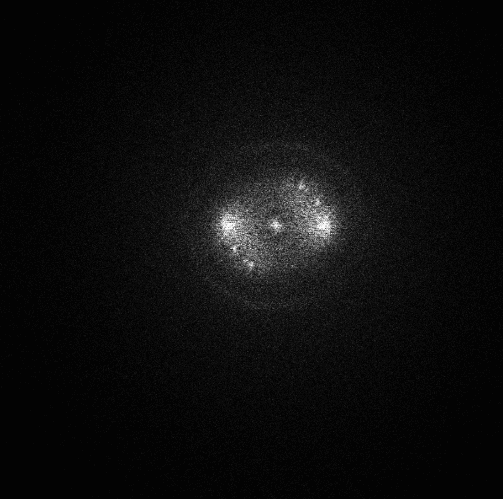

Supplement: S16 Fig — Fast Fourier transform from carbon nanotube in S15 Fig. (TIF) [file pone.0218750.s016.tif]

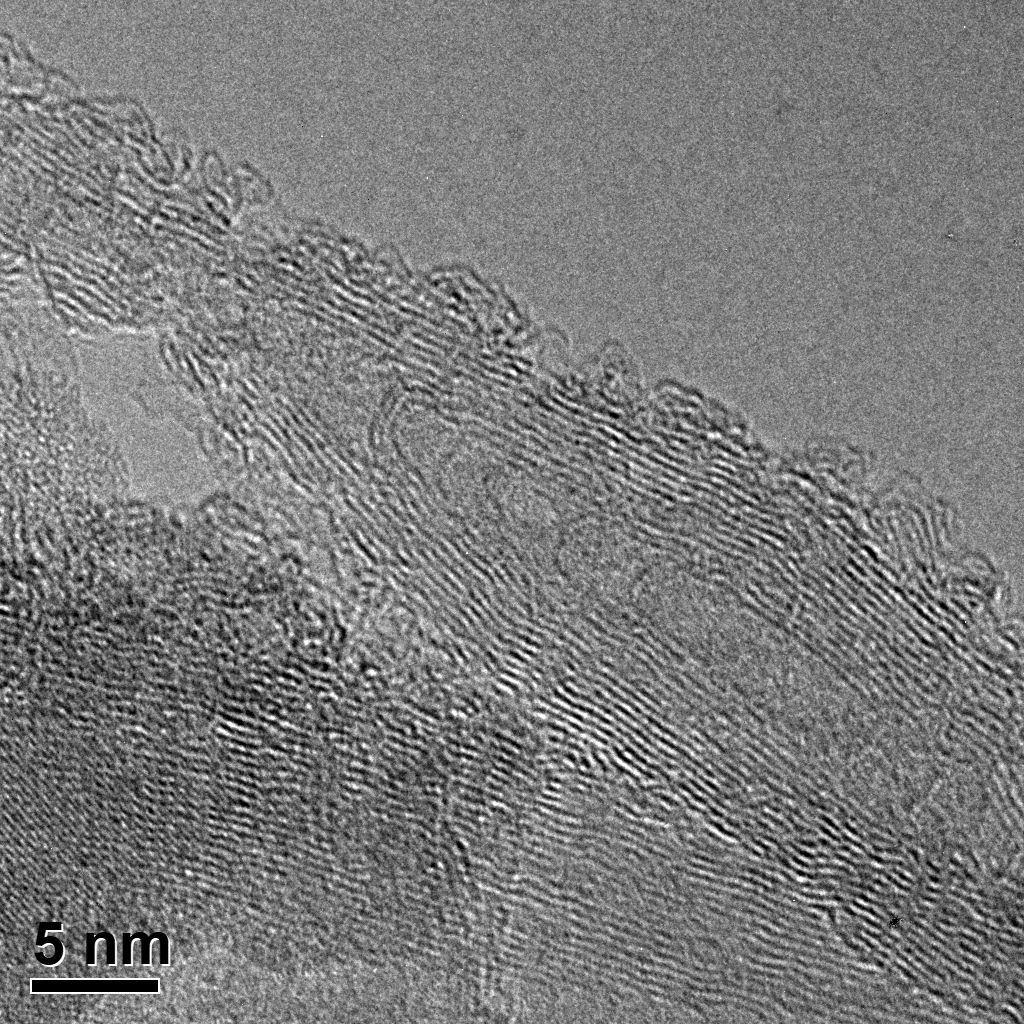

Supplement: S17 Fig — Bamboo-like multiwall carbon nanotube. (TIF) [file pone.0218750.s017.tif]

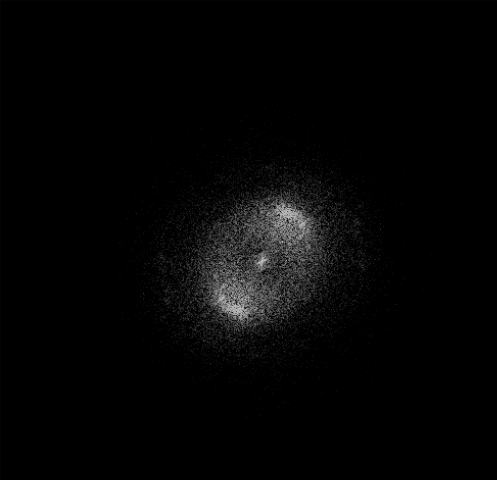

Supplement: S18 Fig — Fast Fourier transform from bamboo-like carbon nanotube in S17 Fig. (TIF) [file pone.0218750.s018.tif]

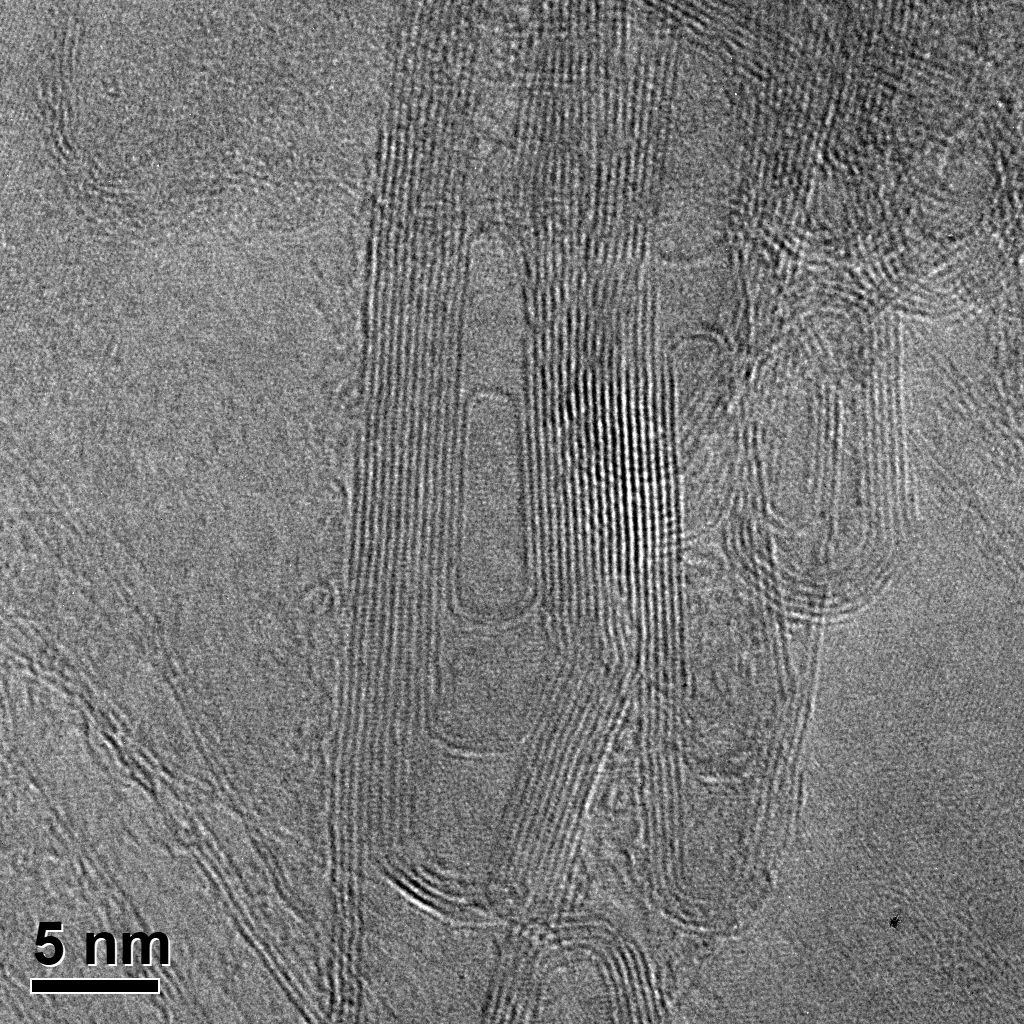

Supplement: S19 Fig — Bamboo-like multiwall carbon nanotube. (TIF) [file pone.0218750.s019.tif]
